# The Effects of Intravenous Fosaprepitant and Ondansetron for the Prevention of Postoperative Nausea and Vomiting in Neurosurgery Patients: A Prospective, Randomized, Double-Blinded Study

**DOI:** 10.1155/2014/307025

**Published:** 2014-06-23

**Authors:** Yasuo M. Tsutsumi, Nami Kakuta, Tomohiro Soga, Katsuyoshi Kume, Eisuke Hamaguchi, Rie Tsutsumi, Katsuya Tanaka

**Affiliations:** ^1^Department of Anesthesiology, The University of Tokushima, 3-18-15 Kuramoto, Tokushima 770-8503, Japan; ^2^Department of Nutrition, The University of Tokushima, 3-18-15 Kuramoto, Tokushima 770-8503, Japan

## Abstract

The incidence of postoperative nausea and vomiting (PONV) is 30–50% after surgery. PONV occurs frequently, especially after craniotomy. In this study, we investigated the preventive effects on PONV in a randomized study by comparing patients who had been administered fosaprepitant, a neurokinin-1 (NK1) receptor antagonist, or ondansetron intravenously. Sixty-four patients undergoing craniotomy were randomly allocated to receive fosaprepitant 150 mg i.v. (NK1 group, *n* = 32) or ondansetron 4 mg i.v. (ONS group, *n* = 32) before anesthesia. The incidence of vomiting was significantly less in the NK1 group, where 2 of 32 (6%) patients experienced vomiting compared to 16 of 32 (50%) patients in the ONS group during the first 24 and 48 hours following surgery. Additionally, the incidence of complete response (no vomiting and no rescue antiemetic use) was significantly higher in the NK1 group than in the ONS group, and was 66% versus 41%, respectively, during the first 24 hours, and 63% versus 38%, respectively, during the first 48 hours. In patients undergoing craniotomy, fosaprepitant is more effective than ondansetron in increasing the rate of complete response and decreasing the incidence of vomiting at 24 and 48 hours postoperatively.

## 1. Introduction

Postoperative nausea and vomiting (PONV) are distressing and frequent adverse effects of anesthesia and surgery. The overall incidence of vomiting is about 30%, while the incidence of nausea is about 50%; in a subset of high-risk patients, the PONV rate can be as great as 70% to 80% [1–4]. Even in patients receiving prophylactic treatment for PONV such as ondansetron, a selective 5-hydroxytryptamine type 3 (5-HT3) receptor antagonist, the incidence of PONV in the first operative day is 30% to 40% [2, 5, 6]. Furthermore, craniotomy surgery leads to changes in intracranial pressure, cerebral intravascular pressure, hemostasis, and cerebral perfusion, resulting in an increased risk of PONV.

Aprepitant, a neurokinin-1 (NK1) receptor antagonist, blockades the central effects of substance P. Substance P is a neurotransmitter found in central areas associated with emesis such as the dorsal vagal complex and area postrema [7, 8]. Recently, we reported that the oral administration of aprepitant effectively diminished the severity of nausea (0–2 hours after surgery) and PONV (2–24 hours after surgery) in laparoscopic gynecological procedures [9]. Fosaprepitant is a new prodrug of aprepitant, which, when administered intravenously, is rapidly converted to aprepitant by phosphatase enzymes [10]. Additionally, the superior efficacy of fosaprepitant for the prevention of chemotherapy-induced nausea and vomiting has been reported to be much longer in duration than other antiemetics [11].

Thus, in this study, we investigated the preventive effects of PONV in a randomized, double-blinded study by comparing the 48-hour postoperative outcomes of patients who had been administered fosaprepitant or ondansetron intravenously prior to craniotomy surgery.

## 2. Methods

This study was approved by the Human Research Ethics Committee of the University of Tokushima and registered in a clinical trials database (UMIN000008621). Written informed consent was obtained from all patients, and the study was carried out in accordance with the principles outlined in the Declaration of Helsinki.

Patients between the ages of 20 and 80 years with an ASA physical status of I-II who were undergoing elective craniotomy under general anesthesia were enrolled in this double-blinded, randomized, controlled study between October 1, 2012, and February 5, 2014. The following exclusion criteria were used for the study: an ASA status of III-IV, neuronal disease, abnormal liver and/or renal function, and patients receiving another antiemetic. All patients were questioned about gender, history of PONV, motion sickness, and smoking status.

The patients received allocations in a randomized, double-blinded manner using a computer-generated distribution (QuickCalcs, GraphPad Inc., La Jolla, CA, USA). To ensure blinding among the investigators, the randomization schedule was generated by a statistician who was not involved in the clinical study. On the day of surgery, patients were randomized to 1 of 2 groups: the NK1 group received i.v. fosaprepitant 150 mg, while the ONS group received i.v. ondansetron 4 mg. The antiemetic was infused over 30 min before anesthesia induction, as indicated in the approved prescribing information for the drugs.

Anesthesia was induced with 0.3 to 0.5 *μ*g/kg/min of remifentanil, 1 to 2 mg/kg of propofol, and 0.8 mg/kg rocuronium to facilitate endotracheal intubation. Anesthesia was maintained with propofol (TCI: 2.0–4.0 *μ*g/mL) in an oxygen and air mixture and 0.1 to 0.5 *μ*g/kg/min remifentanil, and incremental doses of rocuronium were used as necessary for muscle relaxation. Sugammadex 2 mg/kg was used for muscle relaxation reversal at the end of surgery. A rescue antiemetic (10 mg metoclopramide) and/or analgesic was administered at the patient's request.

An anesthesiologist unaware of the patients' randomization collected the data. The incidence of nausea and vomiting, use of rescue antiemetics, and severity of pain were evaluated at 2, 24, and 48 hours after surgery. Patient pain was recorded using a visual analog scale (VAS) pain score (0 = no pain to 10 = the worst pain imaginable). All adverse events were recorded during the first 48 hours postoperatively.

Statistical analyses were performed using Prism version 6.0 software (GraphPad Inc.). Data are expressed as the mean ± SD. Values of *P* < 0.05 were considered statistically significant. For the end points and exploratory analysis, a *χ* ^2^ test was used for analyzing the cumulative incidence of vomiting at each time point, incidence of nausea, use of rescue antiemetics, and complete response (no vomiting and no rescue) for 0 to 2 hours, 0 to 24 hours, and 0 to 48 hours postoperatively. Mann-Whitney *U*-tests were used to analyze VAS pain scores during the same time periods. Kaplan-Meier curves were generated for time to first vomiting during the first 48 hours, and log-rank tests were used to compare treatments.

A previous study reported that oral aprepitant was superior to i.v. ondansetron for the prevention of vomiting, but not for nausea, use of rescue antiemetics, or complete response in the first 24 and 48 hours [12]. As a study using i.v. fosaprepitant has not been previously reported, we estimated the effect size prior to this trial as measured using the same methods based on 10 participants per group to detect a reduction in the incidence of vomiting in the i.v. fosaprepitant group compared to that of the i.v. ondansetron group. A power analysis performed using a test of equality of 2 proportions suggested that 32 patients per group would have an 80% power to detect a 30% absolute reduction in the incidence of vomiting from 40% in the i.v. ondansetron group to 10% in the i.v. fosaprepitant group at *α* = 0.05.

## 3. Results

**Figure 1:**
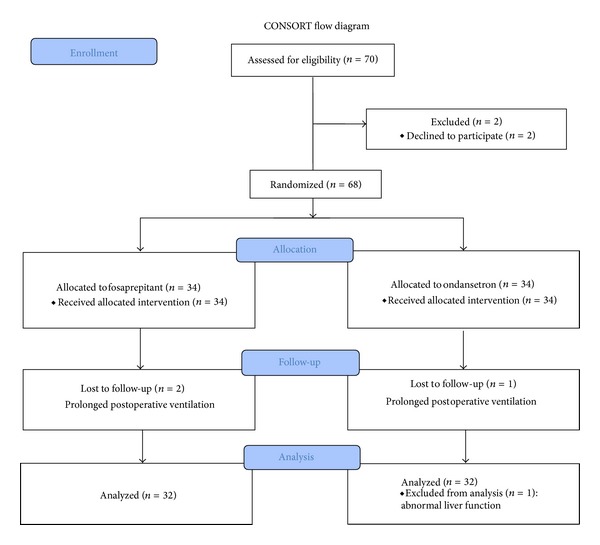
A CONSORT flow chart showing the flow of patients enrolled in the study.

Of 70 randomized patients, 68 received study medications and 64 completed the trial: 32 in the NK1 group and 32 in the ONS group (Figure 1). There was no difference in patient demographics, risk factors for PONV, duration of surgery and anesthesia, intraoperative opioid usage, or postoperative analgesic consumption between the 2 groups (Table 1).

The incidence of PONV and vomiting, the complete response rate, the need for rescue antiemetics, and VAS pain scores were not significantly different between the 2 groups during the first 2 postoperative hours (Table 2).

For the period from 0 to 24 hours, the percentage of patients who experienced vomiting (6% versus 50%, *P* < 0.001, odds ratio = 0.067, 95% confidence interval [CI] 0.014 to 0.327) and the complete response rate (66% versus 41%, *P* = 0.045, odds ratio = 2.790, 95% CI 1.011 to 7.698) were significantly different in the NK1 group compared to the ONS group. However, there were no statistically significant differences between the 2 groups in the incidence of PONV or the need for rescue antiemetics during this time period (Table 2). The incidence of vomiting and complete response from 0 to 48 hours were similar to rates from 0 to 24 hours (*P* < 0.05).

**Figure 2:**
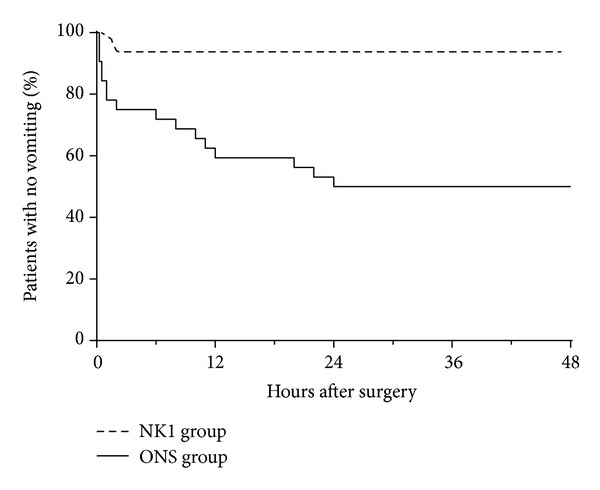
Kaplan-Meier curves for the time to first vomiting during the first 48 hours after surgery. NK1 group = i.v. fosaprepitant. ONS group = i.v. ondansetron.

In the time-to-event analysis of first vomiting within 48 hours, 30 patients were censored at the 48-hour time point. The Kaplan-Meier plot of time to vomiting is shown in Figure 2. The patients in the NK1 group had a longer time to vomiting than patients in the ONS group (*P* < 0.001 based on a log-rank test).

## 4. Discussion

The main findings of the present study were that intravenously administered fosaprepitant can effectively decrease vomiting after craniotomy. For the period between 0 and 2 hours, the number of patients with a complete response and no vomiting did not differ between the groups. However, the intravenously administered fosaprepitant group showed a significantly higher complete response ratio and an effectively lowered incidence of vomiting at the 24-hour and 48-hour time points. These results suggest that an NK1 blockade may be advantageous in suppressing vomiting and may be beneficial if administered prior to this type of surgery.

Substance P is one of the neurotransmitters found in both the central and peripheral nervous systems, and it is known to bind to NK1 receptors. NK1 receptor antagonists also work against both peripherally and centrally induced emesis, although 5-HT3 receptor antagonists have questionable efficacy against centrally induced emesis [13–16]. However, the currently available antiemetics, including aprepitant, do not provide complete protection, and the mechanisms of the presentational effects of PONV are not fully understood.

An NK1 antagonist has already been proven to be an effective treatment for the prevention of chemotherapy-induced nausea and vomiting [17]. Our previous study provided evidence that orally administered aprepitant was also effective in reducing the incidence of these symptoms after surgery [9]. Diemunsch et al. [18–20] compared the antiemetic effects of aprepitant and ondansetron. In their studies, aprepitant was more effective in preventing PONV than ondansetron [18] and aprepitant was significantly more effective than ondansetron for the prevention of vomiting [19]. Gan et al. [12] also determined that aprepitant and ondansetron were similar in their effects on nausea reduction, but aprepitant was more effective in the prevention of vomiting than ondansetron in a study comparing the 2 drugs. Recently, Vallejo et al. [21] reported that the addition of aprepitant to ondansetron significantly decreased postoperative vomiting rates and nausea severity for up to 48 hours postoperatively in patients undergoing plastic surgery. However, the mechanisms of action of intravenous NK1 receptor antagonists such as fosaprepitant in preventing postoperative emesis are not known.

In the present study, we demonstrated that intravenous fosaprepitant was significantly more effective than ondansetron for the prevention of vomiting during both the first 24 and 48 hours after craniotomy. Postoperative vomiting was reduced by 6% in the NK1 group, and these episodes occurred in the first 2 hours. These results are similar to those of previous studies demonstrating a 90% reduction in vomiting with 40 mg of aprepitant as shown by Gan et al. [12] and an 84% reduction as shown by Diemunsch et al. [19]. In addition, the incidence of PONV in our study was 38% in the NK1 group compared to 56% in the ONS group during the first 48 hours (*P* = 0.13). In a similar study by Gan et al. [12], there was an incidence of 50% with aprepitant compared to 43% with ondansetron. When used in combination, aprepitant and dexamethasone are more effective than the combination of ondansetron and dexamethasone in preventing vomiting after craniotomy [22]. However, no difference was observed in the incidence of nausea between the groups in the first 48 hours (69% versus 60%, resp.) [22]. Taken together, we suggest that fosaprepitant is effective in preventing postoperative vomiting and improved efficacy was maintained over 48 hours, consistent with the short half-life of ondansetron and longer duration of action of fosaprepitant; however, fosaprepitant was not more effective in preventing nausea than ondansetron.

Our study has some limitations. Adverse events associated with fosaprepitant and ondansetron are extremely rare and generally minor. Headache and dizziness are the most common known side effects of these drugs. However, in the present study, we could not analyze the incidence of headache and dizziness because of the neurosurgical postcraniotomy status of the patients. One patient in the ONS group had tobe excluded from the analysis because of an adverse event consistent with abnormal liver function, which was not related to the study drug.

Another possible limitation is the dose and timing of the administered drugs. Oral aprepitant doses of 125 mg tended to show a similar reduction in effects compared to oral doses of 40 mg, suggesting a plateau in response; the recommended and approved dose of oral aprepitant for PONV prophylaxis is 40 mg [12, 18]. The intravenously administered prodrug fosaprepitant is converted to aprepitant within 30 min: 115 mg i.v. fosaprepitant is equivalent to 125 mg oral aprepitant [10]. However, the use of fosaprepitant in patients undergoing neurosurgery has not been previously studied. In the current study, the 150 mg i.v. dose was selected, because it is well tolerated and is within the range of previously evaluated fosaprepitant doses [23–25]. Additionally, antiemetics have been shown to be more efficacious when administered toward the end of surgery rather than at anesthesia induction [26, 27]. Further study is needed to characterize the clinical profile of fosaprepitant in other settings such as the treatment of established nausea and vomiting in surgical patients and its potential usefulness in combination with other antiemetics.

## 5. Conclusion

The present study suggests that fosaprepitant showed superiority in complete response and was superior to ondansetron in the prevention of vomiting during the periods 0–24 hours and 0–48 hours after surgery, but no significant differences were observed between fosaprepitant and ondansetron in PONV, in the need for rescue antiemetics, or in the VAS pain scores in patients that underwent craniotomy.

## Figures and Tables

**Table 1:** Patient demographics.

	NK1 group	ONS group
Patients	*n* = 32	*n* = 32
Age, years	62 ± 10	58 ± 14
Height, cm	158 ± 9	158 ± 6
Weight, kg	59 ± 11	62 ± 9
ASA physical state (*n*), I/II	2/30	1/31
Risk factor		
Tobacco use (*n*)	2	4
History of motion sickness/PONV (*n*)	2	2
Women	17	21
Duration of anesthesia, min	460 ± 138	513 ± 166
Duration of surgery, min	366 ± 137	403 ± 197
Intraoperative remifentanil, mg	6.8 ± 4.6	8.0 ± 4.3
Blood loss, mL	291 ± 372	303 ± 287
Fluid volume, mL	3259 ± 1341	3362 ± 1130
Analgesics used postoperatively		
Diclofenac sodium (25 mg sup)	5	4
Pentazocine (15 mg i.v.)	1	1
Loxoprofen sodium (60 mg p.o.)	11	12

Dates are expressed as mean ± SD or number of patients.

NK1 group = i.v. fosaprepitant. ONS group = i.v. ondansetron.

PONV = postoperative nausea and vomiting.

**Table 2:** Postoperative values.

	NK1 group, *n* = 32	ONS group, *n* = 32
0–2 hours		
PONV	8 (25%)	12 (38%)
Complete response	23 (72%)	20 (63%)
Vomiting	2 (6%)	8 (25%)
Rescue antiemetic	6	8
VAS pain score	3 (0–5)	4 (0–6)
0–24 hours		
PONV	11 (34%)	18 (56%)
Complete response	21 (66%)∗	13 (41%)
Vomiting	2 (6%)∗	16 (50%)
Rescue antiemetic	8	14
VAS pain score	3 (0–4.75)	3 (0–4)
0–48 hours		
PONV	12 (38%)	18 (56%)
Complete response	20 (63%)∗	12 (38%)
Vomiting	2 (6%)∗	16 (50%)
Rescue antiemetic	10	14
VAS pain score	2 (0–3.75)	2 (0–2.75)

Dates are expressed as number of patients (percentile) or median (interquartile range). NK1 group = i.v. fosaprepitant. ONS group = i.v. ondansetron. PONV = postoperative nausea and vomiting. VAS pain score = visual analog pain score (0 = no pain to 10 = the worst pain imaginable). *P < 0.05 compared to ONS group.
